# Changes in Microbiome Correspond with Diminished Lung Pathophysiology Following Early-Life Respiratory Syncytial Virus Infection or Antibiotic Treatment: Microbiome Following RSV Infection

**DOI:** 10.3390/v17121632

**Published:** 2025-12-17

**Authors:** Kazuma Yagi, Alexander D. Ethridge, Nobuhiro Asai, Carrie-Anne Malinczak, Llilian Arzola Martinez, Andrew J. Rasky, Susan B. Morris, Nicole R. Falkowski, Wendy Fonseca, Gary B. Huffnagle, Nicholas W. Lukacs

**Affiliations:** 1 Department of Pathology, University of Michigan Medical School, Ann Arbor, MI 48109, USAlarzolam@med.umich.edu (L.A.M.);; 2 Immunology Graduate Program, University of Michigan Medical School, Ann Arbor, MI 48109, USA; ghuff@umich.edu; 3 Mary H. Weiser Food Allergy Center, University of Michigan Medical School, Ann Arbor, MI 48109, USA; 4 Department of Molecular, Cellular and Developmental Biology, University of Michigan, Ann Arbor, MI 48109, USA; 5 Division of Pulmonary and Critical Care Medicine, Department of Internal Medicine, University of Michigan Medical School, Ann Arbor, MI 48109, USA

**Keywords:** microbiome, microbiota, gut-lung axis, respiratory syncytial virus, respiratory virus infection, pulmonary function

## Abstract

Early-life respiratory syncytial virus (EL-RSV) infection has been implicated in long-term pulmonary disease in children. In these studies, neonatal BALB/c mice were infected at day 7 of life, leading to >35% losses in critical lung function, airway mucus metaplasia, and transcriptional hallmarks of mucus hypersecretion four weeks after RSV infection. While EL-RSV minimally reshaped the resident lung microbiota, it led to significant gut dysbiosis, including a long-term reduction of Proteobacteria that can be a source of protective metabolites related to barrier and immune function. Subsequent studies assessing whether a common infant antibiotic (ampicillin) could mitigate EL-RSV-induced lung alterations revealed further severe gut microbiome alterations and, on its own, later in life, recapitulated the full spectrum of RSV-associated alterations in lung function. Metagenomic inference showed that both RSV and ampicillin administered during early life reduced biosynthetic pathways for microbiome-derived metabolites, which are known to reinforce tight junctions, regulate inflammation, and preserve extracellular matrix elasticity. The shared loss of these metabolic programs provides a mechanistic bridge linking distinct early-life exposures to the microbiome changes and airway mechanical deficits later in life. Collectively, the data suggest that RSV and/or antibiotic-triggered gut dysbiosis is the primary insult that likely promotes improper lung maturation/repair through a metabolite-mediated mechanism and may suggest metabolite restoration as a strategy to promote proper developmental lung function.

## 1. Introduction

Respiratory syncytial virus (RSV) is the leading cause of bronchiolitis in children worldwide and infects almost all infants during the first 2 years of life [1,2]. This viral infection increases the risk for recurrent wheezing and later development of hyperreactive airway disease, including asthma that persists for years after the early-life viral infection has resolved [1,2,3,4,5,6,7,8]. Previous studies have reported that risk factors for developing severe RSV infection (RSV-bronchiolitis) are age (<6 months) and preterm birth [9,10,11,12]. Severe early-life RSV infection could interfere with the development of an appropriate host immune response and impact the structural and functional aspects of the lung later in life [13,14,15]. Emerging evidence has suggested that perturbation of early-life gastrointestinal microbiome could be one of the factors that impact appropriate immune system development and predispose to diseases, including severe respiratory infections, asthma, and autoimmune diseases [16,17,18,19,20]. This evidence is supported by the association between early antibiotic use and the direct impact on the microbiome and the development of long-term lung disease, such as childhood asthma [21,22,23,24]. While current recommendations suggest avoiding antibiotic use in infants with bronchiolitis (RSV is the most common cause), recent prospective reviews of antibiotic use suggest that there may be increased use of antibiotics in healthy individuals and those with bronchiolitis [25,26,27,28]. The common mechanism suggested in microbiome influence is metabolites derived from bacteria that have been shown to play an important role in both local and systemic signaling molecules in terms of maintaining immune and tissue homeostasis [29,30]. Several studies also suggested that gastrointestinal microbiome alteration during early life could impact changes in the microbiome-derived metabolites profile, such as short-chain fatty acids (SCFAs) that are implicated in the reduction of airway inflammatory infiltration [31,32,33].

Several epidemiological studies have also demonstrated that environmental factors significantly affect the respiratory tract microbiome and its association with the predisposing conditions to respiratory tract infection [34,35]. A shift in the respiratory microbial composition following respiratory viruses, such as RSV infection, might worsen the disease activity [36,37,38]. Moreover, some studies have indicated that microbial composition could be associated with disease severity in patients with RSV infection and predispose to subsequent development of recurrent wheezing or asthma [39,40,41,42,43,44]. Our recent study has suggested that immune responses and the subsequent development of allergen-induced asthma after early-life RSV infection are associated with changes in the lung and gut microbiomes [45]. The aim of this study is to explore whether early-life RSV infection-induced changes in the lung and gut microbiome could impact pulmonary pathophysiology later in life using an established animal model [46]. Strikingly, ampicillin treatment alone was sufficient to recapitulate the long-term lung function changes observed with EL-RSV infection, supporting our proposed concept that microbiome alteration is sufficient to modify lung function.

## 2. Methods

### 2.1. Animals

All animal studies were approved by the University of Michigan Institutional Animal Care and Use Committee (PRO00010620). Male and female BALB/c mice (6–8 weeks old) were obtained from The Jackson Laboratory (Bar Harbor, ME, USA) and used as breeders to generate experimental cohorts. Neonatal male and female mice bred in-house were used for all experiments. Animals were housed under specific pathogen-free conditions in the Unit for Laboratory Animal Medicine at the University of Michigan.

### 2.2. RSV Infection

A chimeric respiratory syncytial virus (RSV) strain with recombinant Line19 fusion protein was used for all experiments as previously described [47]. Mice were infected intranasally (5 µL/animal) with 2.5 × 10^5^ plaque-forming units (pfu) of RSV A2/L19-F at 7 days of age (RSV group). Viral stocks were grown in Hep-2 cells and concentrations determined by plaque assay. Virus was ultra-centrifuged (100,000× *g* for 30 min at 4 °C) and resuspended in saline prior to use. As for uninfected mice, they were administered 5 µL saline on the same days of age (CTL group). Previous studies have shown that inactivated RSV does not promote airway changes [48]. Mice were euthanized with a lethal injection of sodium pentobarbital and harvested at 8 days and 4 weeks post-RSV infection.

### 2.3. Antibiotic Treatment

Balb/c mice were treated with ampicillin (100 mg/kg/day) at day 15 of life with or without EL-RSV infection on day 7 of life for 7 days. The animals were allowed to age until 35 days of life (28 days post-RSV infection). The analyses on lung function, lung mRNA analyses, and lung/cecal microbiome analysis were then performed on harvested samples as indicated.

### 2.4. RNA Isolation and Quantitative RT-PCR

Lung tissue (the superior lobe of the right lung) was homogenized in TRIzol reagent, and RNA was extracted according to the manufacturer’s instructions (Invitrogen, Carlsbad, CA, USA). cDNA was synthesized using reverse transcriptase (Applied Biosystems, Foster City, CA, USA), incubating at 37 °C for 1 h, followed by incubation at 95 °C for 1 min to stop the reaction. Quantitative real-time PCR (qPCR) was performed using TaqMan chemistry. Control gene transcription of *18s* (Mm03928990) was used for comparison for calculation. Primers were used to measure *Gob5* mRNA as previously described [48]. Relative expression was calculated using the 2^−ΔΔCT^ method. All reactions were run on a 7500 Real-Time PCR System (Applied Biosystems, Foster City, CA, USA).

### 2.5. Lung Histology

Lobes of the right lung were perfused with 10% formalin for fixation and embedded in paraffin. Processed lungs were then cut into 5 micron sections and stained with periodic acid-Schiff (P.A.S., Middleton, WI, USA) or hematoxylin and eosin (H&E, Middleton, WI, USA). Photomicrographs were captured using a Zeiss Axio Imager Z1 and AxioVision 4.8 software (Zeiss, Munich, Germany).

### 2.6. Pulmonary Functional Test

Animals were subjected to a series of pulmonary functional tests (PFT) using FinePointe Pulmonary Function Testing (Data Sciences International, St. Paul, MN, USA). PFT analyses were performed in anesthetized mice after insertion of a tracheal tube to allow mechanical ventilation and pause techniques, as well as functional residual capacity and quasistatic pressure volume maneuvers. Data were accumulated and calculated on each mouse as previously described [49,50].

### 2.7. Processing for Microbiome Analyses

The whole left lung and cecum (with contents) were collected immediately after euthanasia. To minimize cross-contamination during sampling of the lung, a low-biomass tissue, the lung was harvested before the cecum. Instruments were decontaminated between organs and between individual mice by rinsing with ethanol followed by sterile water. For lung processing, excised lungs were placed in tubes containing 1 mL sterile water and mechanically homogenized using a Tissue-Tearor (BioSpec Products, Bartlesville, OK, USA). The homogenizer was thoroughly cleaned and rinsed with ethanol and sterile water between each sample. Procedural controls were included by collecting “blank” homogenization samples after cleaning both prior to and following sample processing. Lung homogenates were stored at −80 °C until genomic DNA extraction. For cecal processing, the cecum was collected with luminal contents intact and snap frozen in liquid nitrogen. Samples were then stored until genomic DNA extraction.

### 2.8. Bacterial DNA Isolation

Genomic DNA was extracted from mouse tissue following homogenization in PowerBead tubes (Qiagen, Hilden, Germany) by the DNeasy Blood and Tissue kit (Qiagen, Hilden, Germany) using a modified protocol previously described [51].

### 2.9. 16S rRNA Gene Sequencing

The V4 region of the 16S ribosomal RNA (rRNA) gene was amplified from each sample with previously published primers [52] and using a dual-indexing sequencing strategy [53]. Sequencing was performed using an Illumina MiSeq platform (Illumina, San Diego, CA, USA) and the MiSeq Reagent Kit V2 (500 cycles) (Illumina, San Diego, CA, USA Cat# MS-102-2003), according to the instructions of the manufacturer with modifications as published previously [54,55,56]. AccuPrime High-Fidelity Taq Polymerase (Life Technologies Cat# 12346094, Carlsbad, CA, USA) was used. PCR was performed using previously described [55,57]. All procedural controls, including elution buffer, isolation controls, homogenization controls, sterile water and a positive control mock community (Zymo Research Cat# D6306, Irvine, CA, USA) were also amplified by PCR. All library preparation steps after genomic DNA isolation were performed by the University of Michigan Microbiome Core.

### 2.10. Analyses of Microbiome Data

16S rRNA gene sequencing data were processed in mothur (v1.42.3) following the MiSeq SOP [56,58]. Sequences were aligned to the SILVA database (v132) using align.seqs, and OTUs were generated at 97% identity using dist.seqs and cluster. Shared community and genus-level phylotyping files were produced with make.shared and classify.otu. Taxonomic assignment was performed using the mothur implementation of the RDP classifier with training set 16 (trainset16_022016.rdp.fasta; trainset16_022016.rdp.tax) [56].

Microbial ecology analyses were conducted in R (v4.2.2) using vegan (v2.5-7) and mvabund (v4.2.1). Contaminant OTUs identified in procedural controls were removed using the prevalence method in decontam (v1.12.0). OTU tables were normalized to percent of total reads, and OTUs present at >0.1% relative abundance were retained for relative abundance and ordination analyses; all OTU meetings in this threshold were included in downstream analyses.

Community similarity was assessed using Bray–Curtis dissimilarity, and alpha diversity was quantified using the Shannon index. Ordination was performed by PCA on Hellinger-transformed normalized OTU tables using Euclidean distances [59]. Differences in community composition were tested by PERMANOVA (adonis2) with 10,000 permutations.

### 2.11. Metagenomic Inference Using PICRUSt2

All OTUs with relative abundance >0.1% were included in PICRUSt2 analysis [60]. Count data for filtered OTUs were used to generate a biom file with bioinformat (v1.20.0). Representative 16S rRNA OTU sequences exported from mothur were de-gapped and pruned to match the filtered OTU set. Functional profiles were inferred using PICRUSt2 (v2.5.0) with default settings following the official tutorial. Predicted pathway abundances were normalized to total inferred abundance per sample and expressed as relative abundance. To facilitate higher-level interpretation, MetaCyc (v26.5) SmartTables were used to map individual pathways to their direct parent terms within the pathway ontology and to aggregate them into “super pathways” [61]. Super pathway abundance was calculated as the summed relative abundance of all pathways assigned to the same direct parent. Differentially abundant functional pathways were identified using LEfSe [62].

### 2.12. Statistical Analysis

Data were analyzed by R (v. 4.2.2) or GraphPad Prism 9 (Insight Partners, New York, NY, USA). Data presented are mean values ± standard error of the mean (SEM). Comparison of two groups was performed with the Mann–Whitney U Test (Wilcoxon Rank Sum Test). All *p*-values were two-tailed, and <0.05 were considered statistically significant. Only significant *p*-values were identified in the figures.

## 3. Results

### 3.1. Early-Life RSV Infection Elicits Airway Mucus Production and Deteriorates Pulmonary Function Later in Life

**Figure 1:**
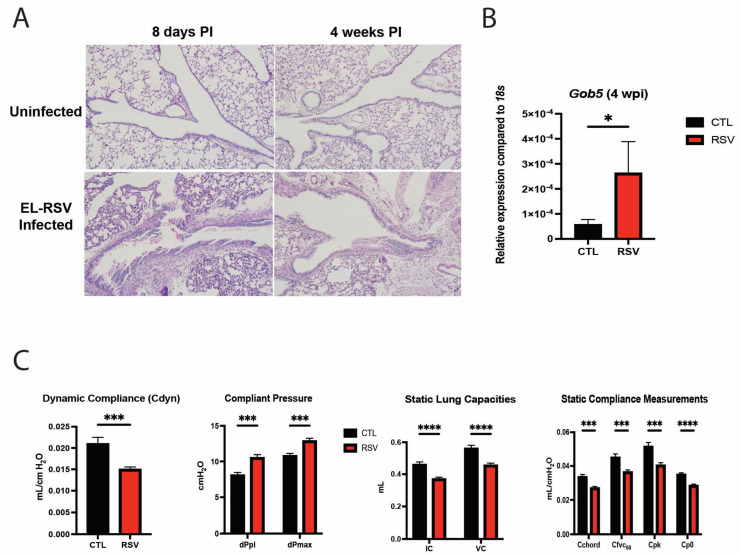
Early-life RSV infection elicited airway mucus production and deteriorated PFT parameters. Neonatal mice were infected with RSV at 7 days of age, and lungs were examined at 8 days and 4 weeks post-RSV infection. The middle and inferior lobes of the right lung were removed and fixed in formalin and embedded in paraffin. Five-micrometer sections were stained with Periodic Acid Schiff (PAS) to visualize mucus deposition. Representative images shown at 200× magnification (**A**). Goblet cell hyperplasia was assessed via gob5 goblet cell gene expression at 4 weeks post-RSV infection (**B**). Data represent mean ± standard error of the mean (SEM). * *p* < 0.05. Neonatal mice infected with RSV at 7 days of age were assessed for pulmonary function tests at 4 weeks post-RSV infection. The measurements taken included static breathing capacities, dynamic breathing capacities, static compliance measurements, and dynamic compliance measurements of the lung (**C**). Data represent mean ± SEM of 12–14 mice/group. * *p* < 0.05, *** *p* < 0.001, **** *p* < 0.0001.

To evaluate the immune response to early-life RSV infection, neonatal mice were infected intranasally with RSV A2/L19-F (2.5 × 10^5^ pfu) at 6 to 7 days of age and harvested at 8 days and 4 weeks post-RSV infection. Histological examination following early-life RSV infection demonstrated an increased number of mucus-secreting goblet cells in the RSV-infected mice compared to the uninfected mice, as indicated by the PAS-stained histopathology examination (Figure 1A). To verify the mucus production within the lungs, *Gob5* gene expression was assessed in lungs by PCR and showed that RSV-infected mice had increased mucus production compared to uninfected mice (Figure 1B, *p* = 0.022). To examine a clinically relevant physiologic parameter, pulmonary function testing (PFT) was utilized at 4 weeks post-early-life RSV infection. Dynamic lung compliance (Cdyn) was significantly decreased in neonatally infected mice, indicative of a stiff lung phenotype (Figure 1C). Measurement of compliant pressure, dPpl and dPmax, representing increased difficulty in breathing, were significantly increased, potentially suggesting remodeling (Figure 1C). We also observed significant decreases in inspiratory capacity (IC) and vital capacity (VC) in RSV-infected mice compared to the uninfected mice (Figure 1C). Furthermore, compliance of the lung was also significantly decreased upon measurement of Chord compliance (Cchord), forced vital capacity (Cfvc50), peak compliance (Cpk) and Cpo (Figure 1C). These studies indicate that there are significant pathophysiologic changes elicited by early-life RSV infection, which could persist for up to 4 weeks post-infection and are associated with pathologic and lung function changes. These studies recapitulate our previous observation regarding changes in lung function and mucus hypersecretion [46].

### 3.2. The Lung Microbiome Is Transiently Altered by Early-Life RSV Infection

**Figure 2:**
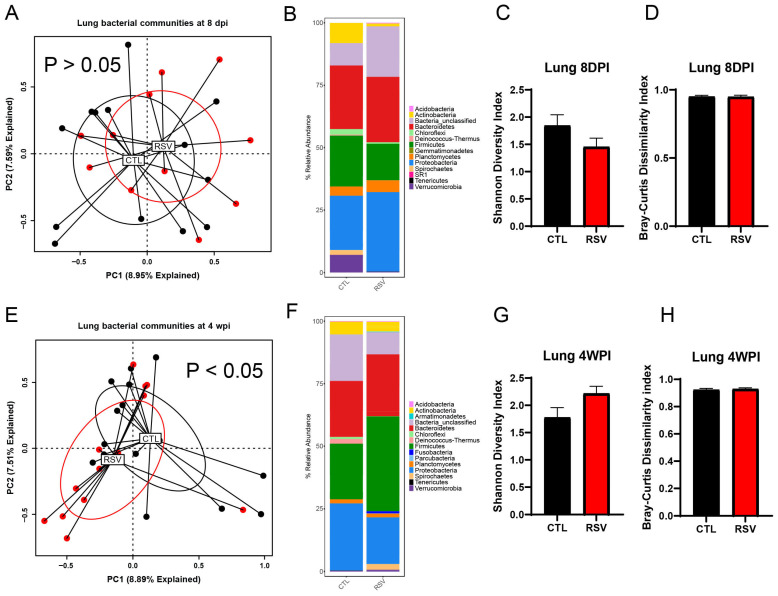
The lung microbiome is altered by early-life RSV infection. The left lungs from uninfected mice and RSV-infected mice were harvested at 8 days and 4 weeks post-RSV infection and processed for 16S rRNA sequencing. The bacterial communities were compared by principal component analysis and PERMANOVA analyses to determine if there were differences in the bacterial communities of the lung at 8 days (**A**) and 4 weeks post-RSV infection (**E**). The bacterial communities in the lungs were also compared by the stacked abundance plots of bacterial phyla at 8 days (**B**) and 4 weeks post-RSV infection (**F**). The bacterial communities in the lungs were further evaluated by Shannon Diversity and Bray–Curtis Dissimilarity Indices at 8 days (**C**,**D**) and 4 weeks post-RSV infection (**G**,**H**) within group and were not significantly different between RSV and control. Data represent 12–14 mice/group.

To explore corresponding changes of lung microbiome following early-life RSV infection, the bacterial communities of the lung samples were profiled using 16S rRNA gene sequencing at 8 days and 4 weeks post-RSV infection. While the overall bacterial composition of the lung was not significantly different between RSV-infected mice and uninfected mice (CTL group) at 8 days post-RSV infection (Figure 2A, *p* = 0.582, PERMANOVA), that of RSV-infected mice at 4 weeks post-RSV infection was significantly different from that of uninfected mice (CTL group) (Figure 2E, *p* < 0.05, PERMANOVA). The variance in the PC1 and PC2 analysis covers 16%, suggesting that infection was not the sole influence on samples. RSV exposure did not significantly affect Shannon diversity or Bray–Curtis dissimilarity indices in lung tissue. Median α-diversity and within-group dispersion were comparable to controls at both 8 dpi and 4 wpi (*p* > 0.05 for all comparisons). Lung bacterial communities in both groups were dominated by Proteobacteria, Firmicutes, Actinobacteria and Bacteroidetes. RSV infection produced a small, non-significant enrichment of Proteobacteria with a reciprocal reduction in Firmicutes at 8 dpi; these differences had largely resolved by 4 wpi with increased Firmicutes and a reduction in Proteobacteria. Thus, in the lung, a transient and subtle alteration was observed in RSV-infected mice.

### 3.3. The Gastrointestinal Microbiome Is Extensively Altered by Early-Life RSV Infection

**Figure 3:**
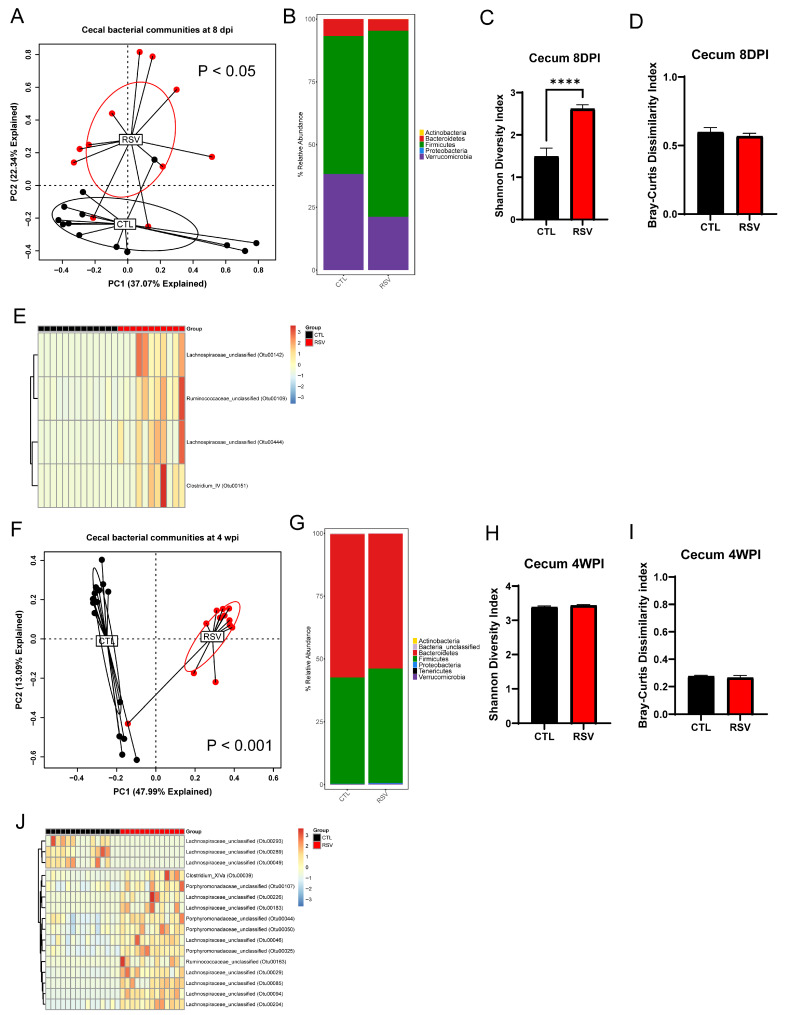
The gastrointestinal microbiome is altered by early-life RSV infection. The cecal samples from uninfected mice and RSV-infected mice were harvested at 8 days and 4 weeks post-RSV infection and processed for 16S rRNA sequencing. The bacterial communities were compared by principal component analysis and PERMANOVA analyses to determine if there were differences in the bacterial communities of the lung at 8 days (**A**) and 4 weeks post-RSV infection (**F**). The bacterial communities in the cecum were also compared by the stacked abundance plots of bacterial phyla at 8 days (**B**) and 4 weeks post-RSV infection (**G**). The bacterial communities in the cecum were further evaluated by Shannon Diversity and Bray–Curtis Dissimilarity Indices at 8 days (**C**,**D**) and 4 weeks post-RSV infection (**H**,**I**). Significant changes (*p* < 0.05) in particular OTUs displayed in the figure were found at both 8 dpi (**E**) and 4 wpi (**J**). Data represent 12–14 mice/group. **** *p* < 0.0001.

The overall composition of the bacterial communities in the cecum of RSV-infected mice (RSV group) was significantly different from that of uninfected mice (CTL group) at 8 days and 4 weeks post-RSV infection (Figure 3; *p* = 0.012 and *p* = 0.001, respectively) as assessed by PERMANOVA. Although there was no significant difference in the Bray–Curtis dissimilarity index between RSV-infected mice and uninfected mice (Figure 3A,F), RSV-infected mice had more bacterial diversity in the cecum analyzed by the Shannon diversity index at 8 days post-infection, but recovered by 4 weeks post-infection (Figure 3C,D,H,I). When examining differences in the stacked relative abundance at the phylum level, it was demonstrated that significant changes in loss of the phylum Verrucomicrobia and Bacteroidetes in the cecum of RSV-infected mice at 8 days post-infection (Figure 3B). RSV animals showed an early increase of Firmicutes with reciprocal loss in Bacteroidetes and Proteobacteria at 8 days post-infection, whereas by 4 weeks post-infection, Firmicutes were suppressed (Figure 3B,G). Linear discriminant analysis revealed consistent depletion of short-chain-fatty-acid–producing *Lachnospiraceae* and *Ruminococcaceae* OTUs and enrichment of multiple *Porphyromonadaceae* members in RSV animals at both 8 days post-infection and 4 weeks post-infection (|log_2_FC| > 1, *q* < 0.05) (Figure 3E,J).

### 3.4. Early Life Antibiotic Treatment Alters Lung Physiology Similar to EL-RSV Infection

**Figure 4:**
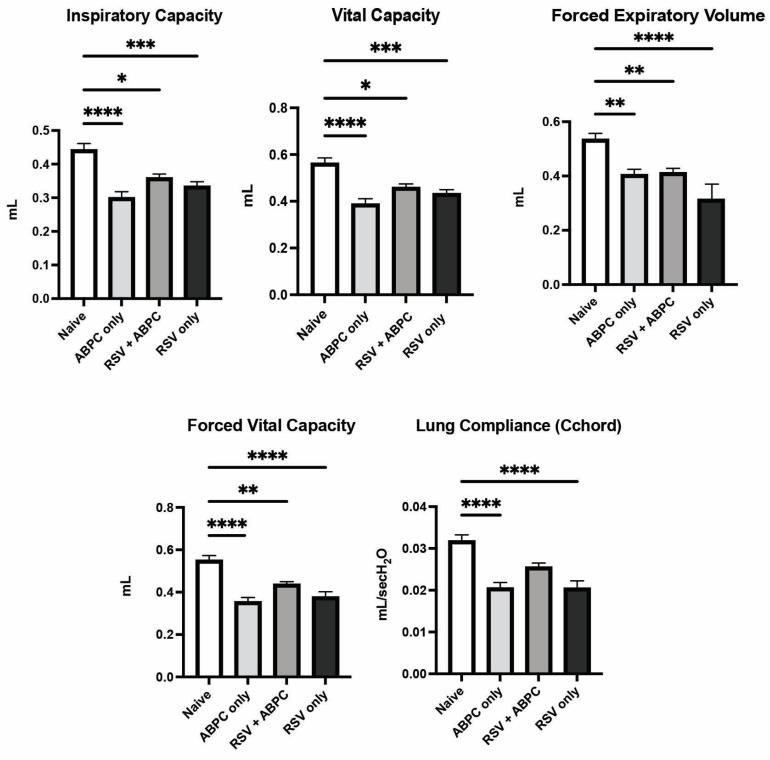
Lung physiology deterioration is induced by both RSV and ampicillin. Neonatal mice infected with RSV at 7 days of age and/or ampicillin (ABPC; 100 mg/kg/day) from day 14–21 of age were assessed for pulmonary function tests at 5 weeks of age (4 weeks post-infection). The altered PFT parameters were assessed in anesthetized mice as described in Materials and Methods. Data represent mean ± SEM of 6–12 mice/group. * *p* < 0.05, ** *p* < 0.01, *** *p* < 0.001, **** *p* < 0.0001.

In order to understand if a clinically relevant treatment regimen of antibiotic to deplete potentially detrimental bacteria could impact the RSV-induced lung function later in life, we utilized ampicillin, which is a commonly used b-lactam antibiotic in infants with pulmonary infections. This antibiotic was delivered systemically by intraperitoneal injection daily from day 8 to day 14 post EL-RSV infection. The lung function changes as in previous studies were examined at 4 weeks post EL-RSV. The EL-RSV induced lung function changes were recapitulated in this experiment as previously identified above, including IC, VC, FEV100, FVC, or Cchord compliance measurements. In animals that received EL-RSV along with ampicillin from day 8–14 post RSV infection, there were similar reductions in lung function measurements (Figure 4). Surprisingly, however, those animals that only received ampicillin but not EL-RSV (i.e., treatment control mice) also showed lung function changes similar to the EL-RSV infected animals (Figure 4). These lung function changes suggest that antibiotic treatment on its own resulted in reduced lung function parameters supporting a role for microbiome in shaping subsequent lung function early in life. 

**Figure 5:**
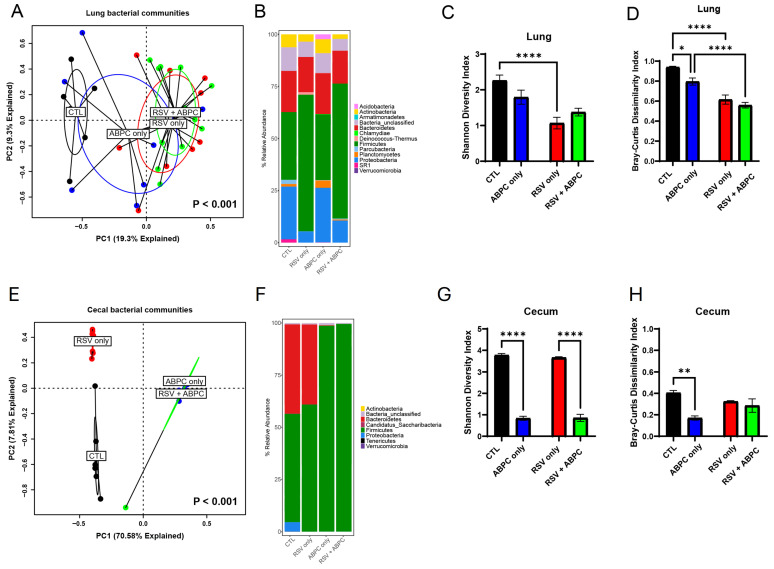
The lung and gut microbiomes are altered by ampicillin with or without RSV infection. The left lungs and cecum from uninfected mice, ampicillin-treated (ABPC; 100 mg/kg/day), and RSV-infected mice were harvested at 4 weeks post-RSV infection and processed for 16S rRNA sequencing. The bacterial communities were compared by principal component analysis and PERMANOVA analyses to determine if there were differences in the bacterial communities of the lung and cecum at 8 days (**A**) and 4 weeks post-RSV infection (**E**). The bacterial communities in the lungs were also compared by the stacked abundance plots of bacterial phyla at 4 weeks post-RSV infection (**B**). The bacterial communities in the cecum were also compared by the stacked abundance plots of bacterial phyla (**F**). The bacterial communities in the lungs (**C**,**D**) and cecum (**G**,**H**) were further evaluated within group by Shannon Diversity and Bray–Curtis Dissimilarity Indices. Data represent mean ± SEM of 6–12 mice/group. * *p* < 0.05, ** *p* < 0.01, **** *p* < 0.0001.

**Figure 6:**
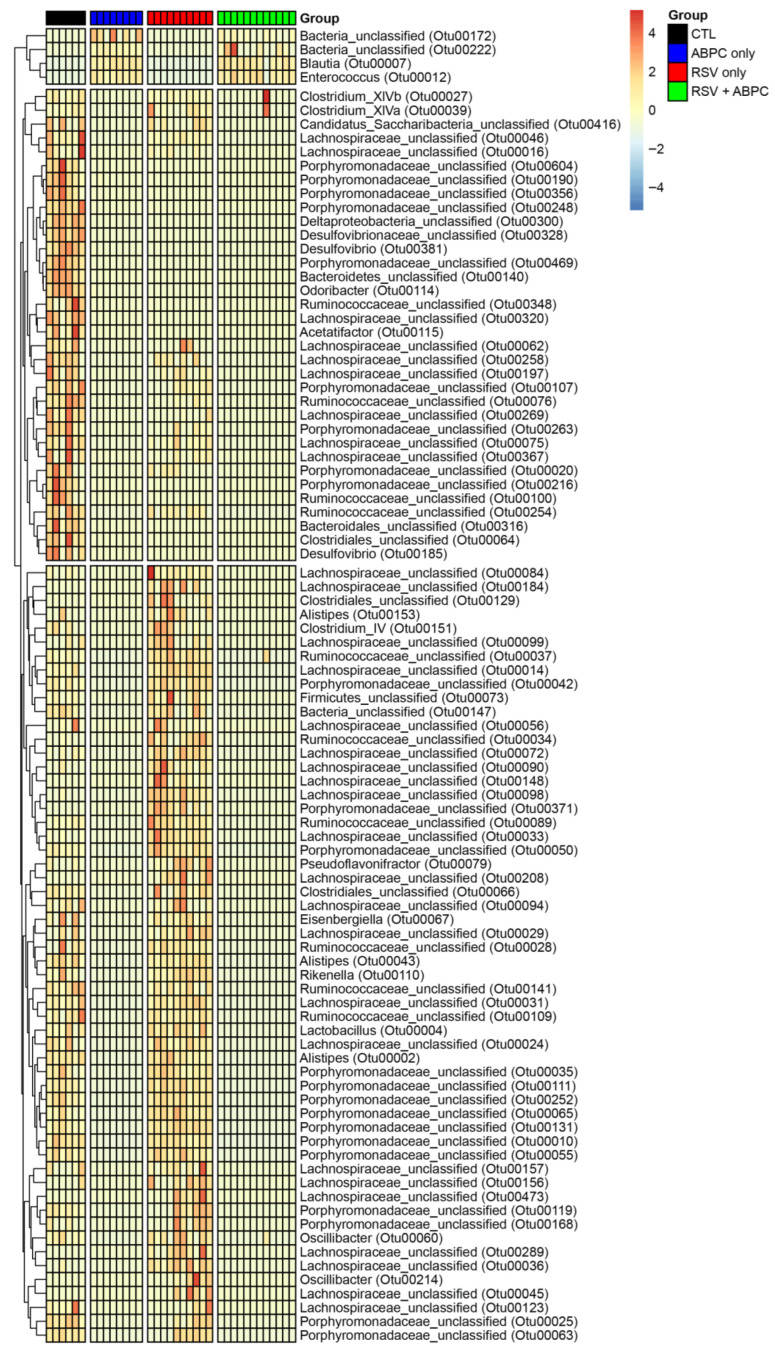
Differentially altered bacteria OTUs in EL-RSV with and without ampicillin treatment. Significantly altered OTUs in the cecal microbiome between treatment groups. OTUs are identified to a family taxonomic level. Values within the heat map represent the fold difference in OTU relative abundance between groups.

In order to better understand whether there was a relationship between the microbiome changes in the ampicillin treated vs. EL-RSV, microbiome analysis was performed at the 4 week post EL-RSV time point in the lung and cecum microbiome of the differentially treated mice. The lung PCA plot of the 4 groups, uninfected control (CTL), EL-RSV, ampicillin only, and EL-RSV + ampicillin showed that while EL-RSV and CTL groups were clearly different the ampicillin only group was somewhat in between and the RSV + ampicillin was very similar to the RSV only group, suggesting that in the lung EL-RSV was dominant (Figure 5A). When examining the Phylum abundance the changes appeared to be most aligned with whether the animals received an EL-RSV infection, with a predominant decrease in the abundance of Proteobacteria (Figure 5B). Although some differential changes could be observed with the ampicillin treated animals compared to untreated controls, the overlap with the EL-RSV treated groups was not obvious (Figure 5B). The Shannon index was not altered by ampicillin in either uninfected or the RSV infected groups. However, the Bray-Curtis Dissimilarity was different in control mice given ampicillin with no infection (Figure 5C,D). When examining the cecal microbiome the PCA plot showed distinct differences between uninfected and the other 3 groups with additional differences determined by ampicillin treatment, whether infected or not (Figure 5E). A consistent loss of Proteobacteria in all 3 treatment groups compared to uninfected animals was the most consistent observation that unified and corresponded to the loss of lung function, while only the ampicillin treated animals (RSV and uninfected) showed a complete loss of Bacteroides Phylum (Figure 5F). The Shannon Diversity Index showed a significant reduction in the ampicillin treated mice in both uninfected and RSV infected animals representing a total collapse of the cecal microbiome (Figure 5G). The Bray-Curtis Dissimilarity Index showed a reduction only in the uninfected animals treated with ABPC (Figure 5H). The analysis of significant changes in specific OTUs in the EL-RSV with or without ampicillin showed that there were common OTUs that were downregulated by EL-RSV infection and ampicillin treatment (Figure 6). Not surprisingly, there were significant numbers of OTUs that were decreased only in the ampicillin treated groups (Figure 6). There were fewer OTUs increased in the ampicillin treated groups, including *Blautia* (Figure 6). 

### 3.5. RSV Infection and Antibiotic Treatment Alter Microbial Functional Potential

**Figure 7:**
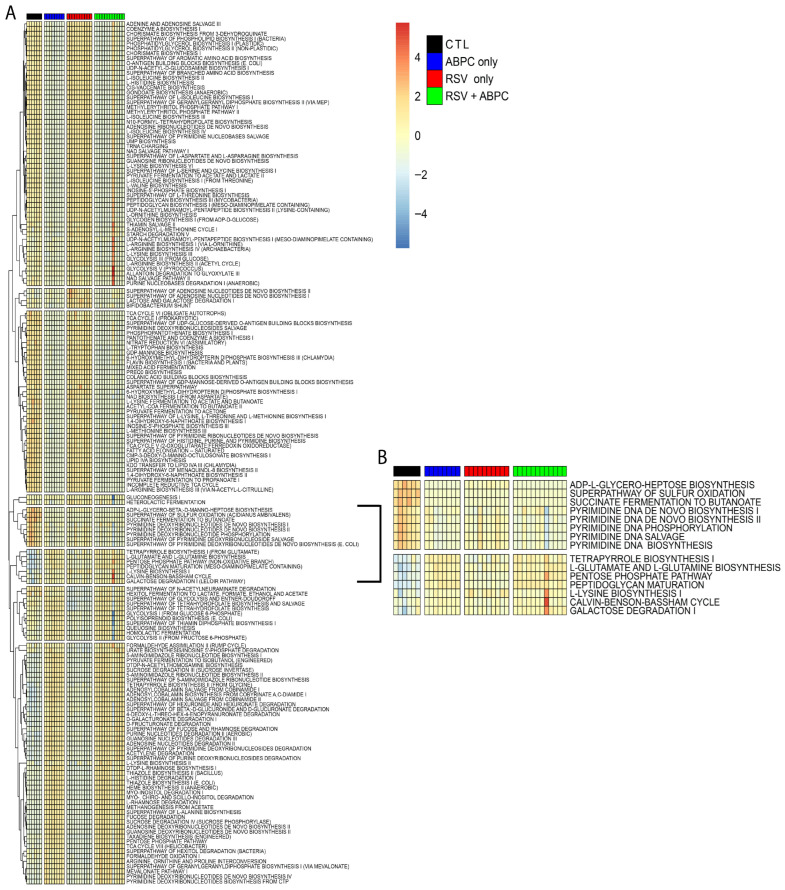
Differentially abundant functional pathways of predicted metagenomes using PICRUSt2 analysis from the cecal bacterial microbiome at 5 weeks of age. (**A**) Hierarchically clustered heatmap of metabolic pathways in cecal bacterial microbiomes at 5 weeks of age for each group as determined by PICRUSt2 and summarized using MetaCyc SmartTables. (**B**) Hierarchically clustered heatmap of significantly different pathway abundances predicted from the cecal bacterial microbiomes that have patterns that correlate to PFT patterns at 5 weeks of age after EL-RSV infection and ampicillin (ABPC; 100 mg/kg/day) treatment. Significant pathways were determined using the LEfSe algorithm with a cutoff of log10 (linear discriminant analysis [LDA]) > 2.

To evaluate how RSV infection and ampicillin exposure affect the functional capacity of the microbiota, we performed predictive metagenomic profiling using PiCRUSt2 on both cecal and lung microbial communities. Heatmaps were generated to visualize predicted microbial metabolic pathways across four experimental groups: control (CTL; 6 mice), ampicillin alone (ABPC_only; 8 mice), RSV alone (RSV_only; 10 mice), and combined RSV with ampicillin (RSV_+_ABPC; 12 mice). The lung microbiome showed only minimal significant functional remodeling as assessed by PiCRUSt 2.0 inference software. In contrast, the cecal microbiome (Figure 7A) displayed pronounced shifts in microbial functional potential in response to RSV infection and antibiotic treatment. In the antibiotic groups, microbial communities showed increased abundance of pathways involved in polyamine biosynthesis, cell structure biosynthesis, and fatty acid and lipid degradation, suggesting an adaptive response to microbiome collapse. These changes were accompanied by consistent suppression of carbohydrate degradation, cofactor biosynthesis, glycan pathways, and energy metabolism, indicating a loss of microbial functions important for gut barrier maintenance and immunoregulation. RSV alone induced broad suppression of biosynthetic and catabolic pathways, reflecting selective pressure on the gut microbiota and loss of metabolic diversity. There were common pathways that were up- and down-regulated in the RSV and antibiotic groups that may have commonly contributed to lung function, including downregulation of DNA synthesis, succinate fermentation, and sulfur oxidation (Figure 7B). The pathways that were commonly upregulated in RSV and antibiotic treatment groups included L-glutamine synthesis, pentose phosphate pathways, L-lysine synthesis and galactose degradation (Figure 7B). Together, these data demonstrate that RSV infection and antibiotic exposure significantly alter the metabolic output of the gut microbiomes, which may contribute to the lung physiology changes observed.

## 4. Discussion

Early-life RSV infection is related to childhood wheezing associated with respiratory virus infection-induced exacerbation, allergies, and asthma, which is accompanied by deteriorated lung function [6,7,8]. Previous epidemiological studies have also reported that early-life RSV changes microbial composition in the respiratory tract and gut of patients and is associated with disease severity [36,42,43,44]. The interaction of responses at different mucosal surfaces has been a focus for many studies, and the dysbiosis of one mucosal organ likely has an impact on responses at other distal mucosal sites [63,64,65,66,67]. In recent studies, it was determined that EL-RSV infection does impact subsequent pulmonary responses upon allergen challenges and also on its own alters long-term pathophysiology, i.e., lung function [46,68,69,70]. Numerous previous studies have outlined that neonatal RSV infection can impact later immune responses and correspond to lung diseases later in life [46,71,72,73,74]. The present study demonstrated that early-life RSV infection elicited airway mucus production and deteriorated pulmonary function later in life, and led to short- and long-term changes in the lung and gastrointestinal microbiome. This study outlines several important aspects that include (1) EL-RSV induced long-term changes in both lung and gut microbiome corresponds to lung function changes, (2) alteration in microbiome on its own by early life antibiotic treatment is sufficient to alter later lung function changes similar to RSV, and (3) lung dysfunction does not need to correlate to an early life infection, rather alteration in lung development is connected to changes in microbiome. Strikingly, the use of ampicillin was enough to alter lung function correlating to clinical observations in asthma studies [75,76,77,78,79], but did not appear to show increased mucus in the upper airways. These salient findings may reflect recent clinical observations in longitudinal birth cohort studies that have observed that early life severe pulmonary infections had a significant impact on lung disease and diminished lung function, asthma, and earlier mortality [80,81,82,83].

Although the overall bacterial composition of the lungs was not significantly different at 8 days post-RSV infection, RSV-infected mice at 4 weeks post-RSV infection showed a significantly different bacterial composition compared to uninfected mice by PCA. The most striking change in the cecum was a significant decrease in the relative abundance of *Akkermansia* in RSV-infected mice at 8 days post-RSV infection. Previous reports have suggested that *Akkermansia* has a protective role in the host by producing SCFAs and other beneficial metabolites, especially early in life [84,85,86]. Fujimura et al. showed that one-month-old infants with low gut Akkermansia levels were more likely to develop severe childhood atopy and asthma [87]. Michalovich et al. also reported that the disease severity of asthma was negatively correlated with the relative abundance of *Akkermansia* in the gut [88]. At 5–6 weeks of age, when *Akkermansia* diminishes in normal mice, the most common effect across the treatment groups in the antibiotic studies was the loss of *Proteobacter* after EL-RSV or ampicillin, and therefore, one might predict that they may have an effect on the changed lung function response observed [89,90]. More extensive analysis of OTUs showed a broad profile of significantly changed organisms that may have impacted not only the development of disease but also the lung development and repair during early life and post-viral processes.

Our findings suggest that early-life RSV infection and ampicillin exposure disrupt the gut microbiome in ways that are likely to undermine lung development/repair and long-term respiratory function. Both insults altered the community of key Proteobacteria and Bacteroides taxa that can be dominant sources of acetate, indole-3-propionate, spermidine, sphingolipids, γ-aminobutyric acid, and other key metabolites that enhance alveolarization, epithelial barrier maturation, and immune tolerance during development and drive epithelial regeneration and junctional stability after injury [30]. PiCRUSt2 inference further revealed a coordinated loss of pathways that synthesize cofactors, process glycans, and fuel energy metabolism, pointing to a microbiome less equipped to support host repair. In parallel, enrichment of polyamine and lipid biosynthetic routes implies a microbiome community that might exacerbate inflammation and inappropriate airway repair. Together, these shifts provide a mechanistic link between antibiotic-altered or RSV-perturbed microbiota and the diminished lung function and heightened asthma risk observed later in life in a clinical setting, and are especially critical early in life [91]. Important future studies should include determining the functional microbiome-fueled metabolites (microbe and/or host derived) that promote proper lung function, as well as expand data related to antibiotics for careful experimental and clinical verification.

## Data Availability

The original contributions presented in this study are included in the article. Further inquiries can be directed to the corresponding author.
